# The Reasons for Physicians and Pharmacists’ Acceptance of Clinical Support Systems in Saudi Arabia

**DOI:** 10.3390/ijerph20043132

**Published:** 2023-02-10

**Authors:** Mohamed Elhassan Seliaman, Mohammed Suliman Albahly

**Affiliations:** Department of Information Systems, College of Computer Science and Information Technology, King Faisal University, Al Ahsa 31982, Saudi Arabia

**Keywords:** health informatics, hospital information systems, clinical decision support systems, user acceptance

## Abstract

This research aims to identify the technological and non-technological factors influencing user acceptance of the CDSS in a group of healthcare facilities in Saudi Arabia. The study proposes an integrated model that indicates the factors to be considered when designing and evaluating CDSS. This model is developed by integrating factors from the “Fit between Individuals, Task, and Technology” (FITT) framework into the three domains of the human, organization, and technology-fit (HOT-fit) model. The resulting FITT-HOT-fit integrated model was tested using a quantitative approach to evaluate the currently implemented CDSS as a part of Hospital Information System BESTCare 2.0 in the Saudi Ministry of National Guard Health Affairs. For data collection, a survey questionnaire was conducted at all Ministry of National Guard Health Affairs hospitals. Then, the collected survey data were analyzed using Structural Equation Modeling (SEM). This analysis included measurement instrument reliability, discriminant validity, convergent validity, and hypothesis testing. Moreover, a CDSS usage data sample was extracted from the data warehouse to be analyzed as an additional data source. The results of the hypotheses test show that usability, availability, and medical history accessibility are critical factors influencing user acceptance of CDSS. This study provides prudence about healthcare facilities and their higher management to adopt CDSS.

## 1. Introduction

One of the most significant causes of healthcare mistakes is the inability to access patients’ medical records due to the lack of implementation of electronic health systems at healthcare facilities [1]. This is a global issue affecting healthcare quality, therefore enhancing patients’ information management would help in improving the quality of health care by reducing medical errors [2]. Although the adoption of Electronic Medical Records (EMR) and other hospital information systems such as Computerized Physician Order Entry (CPOE) has increased [3,4], EMR and CPOE are insufficient to prevent a large number of medication errors without full integration with an intelligent module such as (CDSS) [5,6].

CDSS are specialized information systems used to support several types of clinical decisions. CDSS basically match a patient’s characteristics to a knowledge base and run algorithms to generate warnings, alerts, and recommendations [7]. This definition clarifies the power of integrating patients’ characteristics from EMR with CDSS. Usually, CDSS capture structured data from EMR through CPOE, such as dosage, frequency, duration, and other information [8]. Therefore, some studies categorize EMR and CPOE systems as prerequisites for CDSS [5]. Furthermore, some CDSS are capable of analyzing free text written in EMR, such as a plan of care, admission notes, outpatient clinic notes, and discharge orders [8].

**Figure 1:**
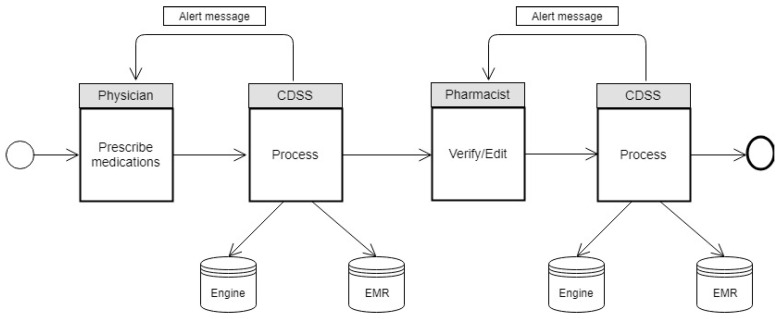
CDSS workflow in BESTCare 2.0.

This research uses BESTCare 2.0 HIS and EMR implemented at the Ministry of National Guard Health Affairs (MNGHA) in the Kingdom of Saudi Arabia as a case study. BESTCare 2.0 is a complete HIS solution, mainly consisting of the following modules: CPOE; registration; scheduling; billing; nursing information system; EMR, pharmacy; laboratory information system; blood bank; medical imaging information system; operating rooms management; infection control; home health care; rehabilitation; Health Information Management; nutrition and food services; and human resources. BESTCare 2.0 is considered an Evidence-Based Patient Care System that utilizes the clinical rules of CDSS and an inference engine to increase patients’ safety and outcomes [9]. CDSS contain a set of defined rules for every medication and check the physician’s entry and compare it with the rules based on the patient’s information in EMR, as shown in Figure 1.

The main issue that healthcare facilities are facing is the adoption and implementation of CPOE and CDSS [10]. Since the physician’s task during medication prescribing is highly complex, the physician must be aware of the patient’s biomedical status, history, and medication interactions and contradictions [11], making prescribing medications a hazardous process. However, the risk could be prevented if physicians and pharmacists received real-time warnings and alerts [12]. Hence, CDSS can be considered a tool to share clinical knowledge among healthcare practitioners to prevent errors and achieve high-quality healthcare [13,14]. Some studies show a high rate of prescription errors, either because of handwritten prescriptions or through EMR, which does not provide real-time clinical alerts [15,16]. Adverse Drug Events have been reported to be at a high rate in multiple hospitals without CDSS [17,18]. Although some healthcare facilities have adopted and implemented CDSS, some studies have shown that certain forms of CDSS have been discontinued [19,20]. The most common reasons for the failure of CDSS are a lack of integration into the business workflow, poor technical support, training issues, and the massive number of prompted alerts, “Alert fatigue” [21,22,23,24]. There is a reported lack of research publications investigating the acceptance of CDSS in Saudi health facilities [25]. Therefore, this research intends to examine the reasons for user acceptance of the currently implemented CDSS as a part of Hospital Information System (HIS) BESTCare 2.0 in the Saudi Ministry of National Guard Health Affairs.

While CDSS have received extensive attention from the research community, this study is motivated by several reasons. First, recent reviews in this domain report an evident necessity for more research to examine the effective adoption of ICT in healthcare in general and the adoption of CDSS in particular [26,27,28]. More specifically, apparent gaps in the knowledge are reported in the research that can identify the organizational, human, and technological factors that might influence the successful implementation of CDSS [25,29,30]. Second, studies on the adoption of CDSS tend to oversee several cultural and contextual aspects that might significantly impact the effective implementation of CDSS [30]. Therefore, it is imperative to replicate and validate studies assessing the adoption of CDSS across different social and cultural contexts [31,32,33]. Third, research in Saudi Arabia is very limited in this domain in general [34]. In particular, research investigating end-user’s acceptance of eHealth services in Saudi Arabia is scarce [35]. Fourth, most of the reviewed conducted studies follow the qualitative method focusing on subjective measures [25,30]. Hence, the rigor of the quantitate method is needed. This research used the Structural equation modeling technique, which is classified as a second-generation statistical method for data analysis. SEM is widely known to be a highly valued recent statistical analysis method, especially for analyzing survey questionnaire collected data [36].

The remainder of this paper is organized as follows: Section 2 presents a theoretical background. Section 3 presents the developed research model and hypotheses, and Section 4 details the research methodology used. Analysis and results are presented in Section 5. A discussion of the study findings is presented in Section 6. Finally, Section 7 concludes the study and highlights its limitations.

## 2. Theoretical Background

This section presents an overview of some prior related research on the adoption and impact of CDSS. The international standards organization defined EMR as “a repository of information regarding the health status of a subject of care in computer process-able form” [37]. EMR systems are designed as integrated modules that cover different types of users, including physicians, nurses, pharmacists, radiology and laboratory technologists, and the hospital’s management [3]. CDSS are defined as integrated information systems that use medical knowledge and provide access to patients’ data and medical history from clinical information systems or electronic medical records to support and enhance the clinical decision-making process [38]. EMR facilitates the physician’s job by providing access to a comprehensive medical repository [39], structured CPOE and forms. The utilization of CPOE’s friendly user interface and CDSS alerts and reminders will minimize medication errors and facilitate monitoring [40]. On the other hand, CDSS and EMR could distract the physician and minimize communication with patients since physicians will spend more time working on these tools [41].

### 2.1. CDSS Adoption and Acceptance Models

Several previous studies have used the Technology Acceptance Model (TAM) and its extended successor models, such as the Unified Theory of Acceptance and Use of Technology (UTAUT), to investigate ICT adoption within the healthcare context [3]. However, there is a lack of research using these technology acceptance models to examine the adoption of CDSS [6].

Some studies show that CDSS have a high failure rate of more than 50 percent [42]. The main reasons for the failures were usability issues [43]. Therefore, the study in [26] conducted a systematic literature review of studies that attempted to assess the implementation of CDSS at healthcare facilities in order to examine the constructs that lead to physicians’ acceptance of CDSS. All factors gathered from the reviewed papers were categorized and mapped to the Human, Organization, and Technology (HOT-fit) framework [44]. (HOT-fit) consists of three main areas or domains: Human, Organization, and Technology. Every domain contains some dimensions. The review found that the System Use dimension was the highest reported by 22 papers, while 20 papers were linked to the System Quality dimension, and 18 papers mentioned Information Quality. The most frequently mentioned factors are ease of use, flexibility, system messages, and user interface design. On the other hand, the least mentioned dimensions were User Satisfaction and Organizational Environment, with three papers each. This systematic review showed that the factors associated with the Technological and Human domain are the most effective in CDSS acceptance. In addition, several human domain factors, such as training, trust, security, and safety, have been found to influence acceptance of CDSS [3].

The “Fit between Individuals, Task, and Technology” (FITT) framework assumes that ICT adoption in the healthcare environment depends on how it fits between the characteristics of systems users, technology features, and the organization [45]. The research in [46] applied the FITT framework to a fully-integrated health electronic service called “HYGEIAnet”. HYGEIAnet is a network of hospital information systems, primary care information systems, and emergency information systems implemented in extensive health facilities and primary care units on the Greek island of Crete [47,48]. This case study aims to find the factors that might influence the adoption of IT services throughout a distributed health environment and show FITT’s applicability to explain the successes and failures of implementation. The research team used quantitative and qualitative methods, including extracted data, interviews, documents review, and site observation. Healthcare practitioners found that the system facilitates their job in terms of retrieving patients’ data and monitoring them, leading to an increase in adoption. The integrated hospital information system consists of a clinical information system, nursing records, medical laboratory information system (LIS), electronic health records (EHR), and pictures archiving and communications systems (PACS). After case analysis, the overall success of the previously mentioned systems was due to some initiatives covering FITT factors from the implementation team. The main initiatives, such as on-job training, 24 h hot-line support, pilot deployments, and managerial support, played a significant role in achieving a high success rate.

### 2.2. CDSS Impact

The success of CDSS can be measured by their impact in minimizing prescription errors. Prescription errors are “any preventable event that may cause or lead to inappropriate medication or patient harm when the medication is in the control of the health care professional, patient or consumer” [49]. Medication errors have been categorized into the four following categories: Serious error (Type A), Major error (Type B), Minor error (Type C), and Trivial error (Type D) [50]. An investigation has been conducted to analyze handwritten medication errors at 10 Primary Healthcare Centers in Riyadh from the public and private sectors [15]. The research team collected paper medical records for 1182 patients from public primary care centers and 1200 from private primary care centers. This research revealed a high error rate (near 1/5), posing a significant threat to patients. Since this paper is limited to the primary care environment, a higher rate of errors is expected at more complicated facilities such as those that provide emergency and critical care services. CDSS can be an excellent solution to reduce the high rate of medical mistakes, even in stressful circumstances [51]. Moreover, most of the studies that discussed the net benefits of CDSS focused on the physician’s practice. Therefore, more research is needed to investigate the benefits of CDSS in terms of minimizing errors and increasing efficiency and effectiveness [25].

## 3. Research Model and Hypotheses

**Figure 2:**
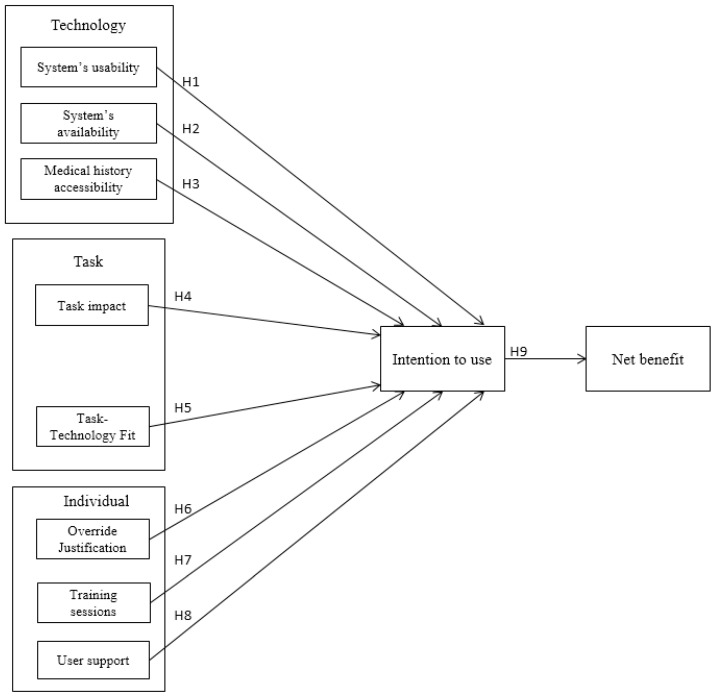
The research model with the hypotheses.

A research model has been developed as a result of integrating the FITT framework into the Hot-fit model. This FITT-HOT-fit integrated model is used to analyze the factors of user acceptance of CDSS. The developed model utilizes the three domains of the FITT framework: technology, task, and individual, in addition to the influence of different domain factors on the adoption of CDSS from the Hot-fit model. The model consists of eight independent variables, one mediating variable, and one dependent variable, as depicted in Figure 2. The description of these variables is as follows.

The system’s usability is defined as the extent to which the users find the system friendly and accessible [52]. For the system users, it means that they find this system easy to use, hence it will support them in performing their tasks without extra effort. This attribute influences the fit between individuals and technology. Evidence from previous research in this area shows that more than half of health information systems fail due to usability issues [53]. Therefore, we state the following hypothesis:

**H1.**  *System’s usability has a positive influence on intention to use.*

The system’s availability is defined as the correct technical functioning of the system [54]. The system should be available and accessible anytime and anywhere within the organization. Otherwise, the tasks will not be performed in the required time. This attribute influences the fit between individual and technology and between task and technology. Hence, we hypothesize:

**H2.**  *System’s availability has a positive influence on intention to use.*

Medical history accessibility can be defined as the completeness, accuracy, organization, currency, and timely availability of patients’ medical history provided in the system to allow health practitioners to obtain information about any of their intended objectives [55]. Since being able to utilize a patient’s medical history is vital to support decision making and avoid any order duplication, it should be accessible and clearly stated in the system. This attribute influences the fit between the users and the technology and between job tasks and the technology. Therefore, one hypothesis related to medical history accessibility is identified as follows:

**H3.**  *Medical history accessibility has a positive influence on intention to use.*

Task impact defines the users’ perceptions about how the system allows them to complete their tasks effectively and improve their work [56]. The users feel that the system allows them to accomplish more work than would otherwise be possible. This attribute influences the fit between individual and task. Therefore, the following hypothesis is stated:

**H4.**  *Task impact has a positive influence on intention to use.*

Task-Technology Fit indicates how the system assists users in performing their work or coursework [57]. The user finds that the system’s functions fit the requirements of tasks or coursework. This attribute influences the fit between the task of users and technology.

Thus, the following hypothesis related to Task-Technology Fit construct is identified as follows:

**H5.**  *Task-Technology Fit has a positive influence on intention to use.*

Training sessions construct is defined as the extent to which an individual has been trained about the system through courses, training, manuals, and so on [52]. This attribute influences the fit between individual and technology. The lack of training is reported as an obstacle to using CDSS for supporting healthcare decisions [58,59].

User support is defined as the perception of how the system’s provider delivers the service to the user [52]. The user will be more satisfied when the provider solves the system’s issues rapidly. This attribute influences the fit between individual and technology.

Override Justification is defined as the reason for rejecting a system’s alerts [60]. This attribute influences the fit between individual and technology. Findings from IS research suggest that physicians will accept systems that allow them to have professional autonomy and practice individual judgment [61]. Therefore, we state the following:

**H6.**  *Override justification positively influences intention to use.*

Intention to use is defined as the user’s intention to use the system [62]. The user is willing to let the system assist him or her in deciding which medication to prescribe.

Net benefit is defined as the benefits of the system as perceived by the user [63]. The system reduces the time and effort required to support decision making.

Therefore, three hypotheses related to the individual are identified as follows:

**H7.**  *Training sessions have a positive influence on intention to use.*

**H8.**  *User support has a positive influence on intention to use.*

**H9.**  *Intention to use has a positive influence on Net benefit.*

## 4. Research Methodology

The research methodology followed in this research is a quantitative approach to achieve the research goals. A survey questionnaire is developed as the main measurement instrument to collect the health practitioners’ responses measuring their behaviors towards the implemented CDSS. The survey questionnaire is used because it has many advantages in IS research. These advantages include the ease of reuse, comparing different perspectives, the capability of predicting behaviors, and the capability of testing types of theoretical propositions objectively [36]. The survey questionnaire provides a clear picture of health practitioners’ experience with such systems. In addition, this study presents the CDSS alerts that popped up for the health practitioners during the medication prescribing process in EMR and their actions and behaviors towards these alerts. The medication prescribing process requires the physician’s order and the pharmacist’s verification or change. The research team used Oracle Data warehouse for extraction and Tableau for visualization. There were five phases to the study implementation: understanding the business workflow; identification of the scope of the required data; data extraction; data modeling; identification of dimensions and measures; and dashboard design.

### 4.1. Survey Design and Instruments Development

The survey design process started with a review of the related literature to find suitable survey questionnaire items for each model construct. After identifying the questionnaire items, the survey was designed and sent to six domain experts (University professors of Computer Science and Information Systems) to test its face and content validity. The experts thankfully provided the researchers with some notes to enhance the survey questionnaire. In addition, the survey evaluation process continued with a pilot study administered to some users chosen randomly to evaluate the questions in terms of clarity, precision, and time taken to complete the survey. Moreover, the data used in this study was collected from five hospitals belonging to the Ministry of National Guard. The hospitals are located in Riyadh, Jeddah, Ahsa, Dammam, and Madinah. The researchers assumed that the questions were suitable for all hospitals as the system was standardized and follows the regulations and legislation of the ministry. The users received the same training materials and support process. After, the survey was written in the English language and published online, and a notification was sent to around 350 users through the department’s managers. The scope of this survey includes physicians and pharmacists from all experience levels. Appendix A shows the latent construct items. A five point Likert scale [64] with anchors of strongly disagree to strongly agree was used to measure each item. The other part of this research, Datawarehouse (DW) data extraction, was conducted at King Abdulaziz Hospital, Al Ahsa, Saudi Arabia, between January 1 2018 and December 31 2018. Physicians and pharmacists from all medical departments and with different levels of experience were included in this study.

### 4.2. Sample Demographic Characteristics

A final sample size of (116) responses is used in this research with resampling. Table 1 shows the basin demographic characteristics of the respondents’ sample: gender, age group, job title, and hospital. Male respondents (85%) reflect the Saudi workplace culture that involves males more than females. We expected most of the respondents to be from Riyadh (56.7%) as King Abdulaziz Medical City in Riyadh is the largest healthcare facility among the National Guard facilities. Jeddah, Madinah, and Dammam respondents were the least. The majority of collected responses are from the Central and Eastern regions.

## 5. Analysis and Results

This research used Analysis of a Moment Structure (AMOS 21.) software to analyze the research model. AMOS is used to assess the psychometric properties of the measurement model and estimate the parameters of the structural model. AMOS enables the simultaneous analysis of indicator variables, allowing an examination of the extensive interactions among latent and moderating predictor variable indicators [65].

### 5.1. The Measurement Model

Reliability results are shown in Table 2. The results indicate that the measures are robust in terms of internal consistency reliability, as indexed by the composite reliability [66]. The composite reliabilities of the measures range from 0.73 to 0.98, exceeding the recommended threshold value of 0.70. In addition, Cronbach’s alpha (CA) has been calculated to assess the reliability of the constructs. The acceptable score of CA is >0.7. However, 0.6 is acceptable if the constructs pass the validity tests [67]. Cronbach’s alpha is significant for all constructs and ranged from 0.78 to 0.95, except for override justification, which scored Cronbach’s alpha of 0.6.

Construct validity is “the extent to which a measure assesses the construct that it is intended or supposed to measure” [68]. Confirmatory Factor Analysis (CFA) is used in this study to assess validity. The assessment includes convergent validity and discriminant validity. Convergent validity can be achieved if each construct’s Average Variance Extracted (AVE) is >=0.5 and the composite reliability is >=0.7. Discriminant validity can be achieved if the square root of AVE of each construct is higher than the inter-construct correlations with all other constructs [68].

Table 3 presents a convergent validity assessment by calculating composite reliability and AVE. AVE for all the variables exceeded 0.50.

Discriminant validity is assessed by computing the square root of AVE for each construct and then comparing these values with the constructs’ correlations. Table 4 presents the discriminant validity assessment results. These results show that all constructs passed the test. The square root of AVE must be higher than all constructs’ correlations with other constructs [68].

Factor loadings for each variable must be at least 0.5, or the variable becomes a candidate for deletion [69]. The factor loadings were calculated using AMOS for each construct. Table 5 shows the results from a lower threshold of 0.544 to an upper threshold of 0.958. The results show that the factor loading for each item is highly significant (*p* < 0.001).

### 5.2. The Structural Model

**Figure 3:**
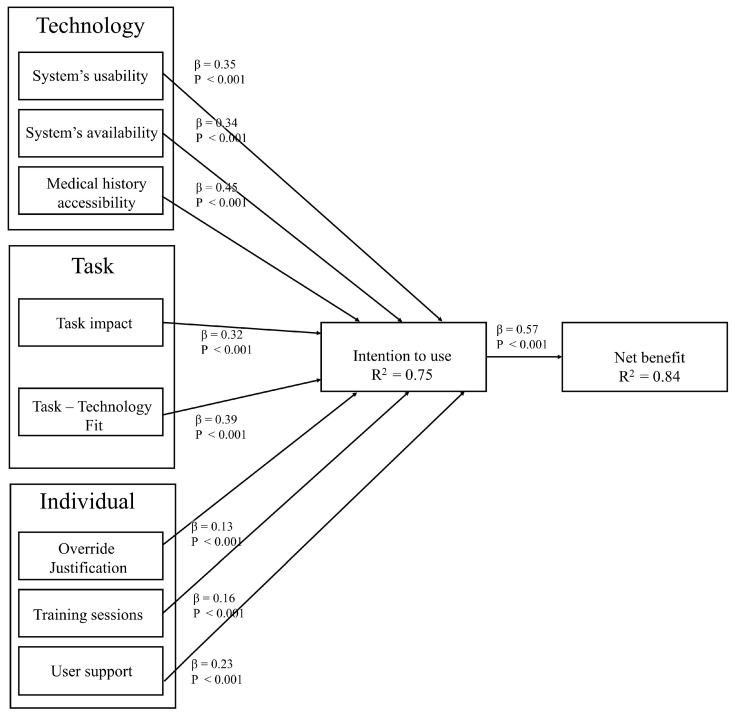
The structural model.

The structural model is a technique used to analyze the relationships between latent constructs and measured variables. Figure 3 shows the structural model results. All beta path coefficients are positive and statistically significant (at *p* < 0.001).

In the structural model, the value R2 is the square of the correlation between the predicted values and the observed values, and it indicates the percentage of variation explained by the regression line out of the total variation. Compared to prior models of CDSS acceptance, the model reported better explanatory power of variance in behavior intention to use CDSS. While the model in [70] explained 28% of the variance and the model in [71] explained 47% of the variance, the explanatory power of the model developed in this study is 75% of the variance in behavioral intention. Moreover, the model explained 84% of the variance in perceived net benefits. The value β is the correlation coefficient between two variables. The path coefficient β indicates the magnitude of the independent construct’s effect on the dependent construct. Table 6 presents the hypothesis testing results.

First, we tested the impact of the technology-related factors on the intention to use. Hypothesis 1 states that the system’s usability positively influences the intention to use. A positive path coefficient (β = 0.35, *p* < 0.001) supports hypothesis 1. The system’s availability was hypothesized to affect the intention to use positively. As predicted, it was supported (β = 0.34, *p* < 0.001). Hypothesis 3 states that medical history accessibility positively influences the intention to use. A positive path coefficient (β = 0.45, *p* < 0.001) supports hypothesis 3. Therefore, among the three technology domain-related factors, medical history accessibility reported the highest path coefficient value, indicating this construct’ significant effect on health professionals’ behavioral intention to use CDSS. This result is supported by the lower override rates obtained from the actual CDSS usage data.

Second, two task domain-related factors were examined in the model: Task impact and Task-Technology Fit. Among the two, Task-Technology Fit reported a higher effect on health professionals’ behavioral intention to use CDSS. Task impact was hypothesized to affect the intention to use positively. A positive path coefficient (β = 0.32, *p* < 0.001) revealed that hypothesis 4 was strongly supported. Hypothesis 5 states that Task-Technology Fit positively influences intention to use. As proposed, the hypothesis was supported (β = 0.39, *p* < 0.001).

Third, the model examined three factors related to the individual domain. Hypothesis 6 stated that Override justification positively influences intention to use. A positive path coefficient (β = 0.13, *p* < 0.001) supports hypothesis 6. Training sessions were hypothesized to affect the intention to use positively. A positive path coefficient (β = 0.16, *p* < 0.001) revealed that hypothesis 7 was strongly supported. Hypothesis 8 states that user support positively influences intention to use. A positive path coefficient (β = 0.23, *p* < 0.001) supports hypothesis 8. Finally, hypothesis 9 stated that intention to use positively influences net benefit. A positive path coefficient (β = 0.57, *p* < 0.001) supported hypothesis 9. It can be observed that the three factors related to this domain reported the lowest effect on health professionals’ behavioral intention to use CDSS.

### 5.3. CDSS Usage Data Analysis

In addition to the survey questionnaire data, a sample of 46,212 medication alerts was extracted from the actual system usage history. The most occurred alerts are related to (Single Dose Maximum) and (Drug and Drug Severity Major) as shown in Table 7.

In order to explore the physician’s behavior during different cases and situations, a new important attribute, visit type, was added to this descriptive study. Table 8 shows the total and percentage of alerts and physicians overridden by visit type. As shown in Table 8, the physicians show a high acceptance rate for the received alerts. In the most critical area of any hospital, the ER, physicians accepted (78.14%) of the alerts and overrode (21.86%). Similar results occurred at inpatient and outpatient, with (74.18%) and (71.17%) of the alerts accepted by physicians, respectively. These override rates are meager when compared to high-alert override rates ranging between 49% and 96%, as reported in recent studies [70,72].

## 6. Discussion

This study examines how certain variables affect healthcare practitioners’ intention to use CDSS and how these factors affect their performance and clinical decisions. The researchers developed a model based on the integration between FITT and Hot-fit models by incorporating some additional variables.

The technology variables: usability, availability, and medical history accessibility were found to be important factors to accept using CDSS.

First, we asserted that usability has a positive influence on the intention to use CDSS. The extensive use of questionnaires to examine CDSS usability and user satisfaction is crucial for integrating user feedback into the CDSS development process [73]. The results support this hypothesis. This finding is consistent with observations made by previous research investigating the acceptance of CDSS in other countries [74]. However, the result conflicts with the findings of [33], in which no significant effect of effort expectancy on intention when considering user experience as a moderating variable was found when investigating Saudi users’ acceptance of IT. This might be because the users’ interaction with CDSS is different from their interaction with other information systems.

Second, we hypothesized that the system’s availability has a positive influence on the intention to use CDSS. The results indicate that system’s availability has a significant impact on user acceptance of CDSS. Prior research has shown that system availability has a significant positive impact on the perceived quality of healthcare systems [75]. In turn, perceived quality is a significant predictor of CDSS acceptance.

Third, as expected, the results show that medical history accessibility and system quality positively influence the intention to use CDSS. This finding is consistent with some prior research [75,76,77,78,79].

The model’s second domain covers human factors. This domain covers the constructs: override justification, user training, and technical support variables. This study’s results show that override justification positively influences the intention to use CDSS. Although several previous studies have examined factors influencing the acceptance of CDSS alerts [60], our study is the first attempt to examine the association between override justification and alert acceptance. Physicians think they should be allowed to override the CDSS recommendations and provide supporting evidence [71]. If there is an option to override CDSS alerts, physicians may consider CDSS as a real threat to their professional autonomy [71]. Previous studies show that if physicians consider CDSS a real threat to their professional autonomy and individual judgment, their acceptance of CDSS will be impacted negatively [31]. Actual usage data extracted from the BESTCare 2.0 data warehouse shows that physicians accepted (78.14%) of the alerts and overrode (21.86%) in the ER. Similar results occurred at inpatient and outpatient since (74.18%) and (71.17%) of the alerts were accepted by physicians, respectively.

User training is considered the first contact between the healthcare practitioner and the system. A qualified instructor who has a knowledge of the system’s functionalities, clearly designed material and documents, and a learning management system were the main objectives of MNGHA to be accomplished through the training process. We asserted that training sessions have a positive influence on the intention to use CDSS. The results support this hypothesis. This finding is consistent with other studies [80]. In [81,82], the research team found that training has a positive impact on intention to use the system. Therefore, it is essential to consider the amount of training needed before implementing CDSS [80]. Moreover, the study in [83] conducted a qualitative study that suggested certain organizational characteristics, such as training, are influencing the use of CDSS. As a result, (56%) of survey questionnaire respondents accepted the training approach followed by MNGHA, (21%) did not like it, and the rest were neutral.

Another critical factor is the technical support provided by the Information Systems and Informatics Department (ISID) to the end-users after the system’s implementation. The results show that user support positively influences the intention to use CDSS. Based on the survey results, more than (45%) of the respondents consider ISID employees able to solve technical problems and help them as a courtesy. However, (18%) of the respondents disagree.

The third domain of the developed model covers task variables that measure the impact of system adoption on healthcare practitioners’ daily tasks and productivity. The results support the assertion that task impact significantly positively affects physicians’ intention to use CDSS. This finding is in line with results in prior research in general [31,84,85,86]. In particular, this result validates the finding of the qualitative analysis in [87]. This result can be interpreted by the fact that (70.5%) of the respondents declared that the system helped them to meet patients’ needs, and (74%) of them stated that the system allows them to accomplish more work than before. Additionally, actual usage data extracted from the DW shows that consultants faced 5796 alerts out of 160,725 orders (3.60%), which are classified as the best performance among all job titles. Staff physicians prescribed 466,158 medication orders and received 25,138 alerts (5.40%). Finally, residents with five years or less of experience were prescribed 205,455 orders, and there were 15,233 alerts (7.41%) generated for them. Results from prior research indicate that system usability as measured by effort expectancy has a less significant effect than task impact as measured by performance expectancy on use intention [31,74]. However, our results show that CDSS usability (β = 0.35, *p* < 0.001) is more significant than task impact (β = 0.32, *p* < 0.001). This might be because most of the respondents are willing to use the system to help them through the decision-making process and assist them in choosing the most appropriate medication for each case. Moreover, a significant impact of the system on healthcare practitioner’s performance and patient outcomes has been proven, since the results show that the system reduced the time and effort taken by healthcare practitioners to accomplish their work and make clinical decisions.

Concerning the perceived net benefits of CDSS, most of the studies that discussed CDSS net benefits focused on physician’s practice. Minimal research discussed the net benefits of increasing CDSS efficiency and effectiveness [25]. The current study shows a significant positive correlation between CDSS acceptance and net benefits (β = 0.57, *p* < 0.001). Thus, when CDSS users realize the net benefits of CDSS and believe that the system has changed their job significantly, they are more likely to accept the system.

The successful design and implementation of CDSS requires careful consideration of these three mentioned domains to shorten the treatment process and minimize the time the health practitioner spends performing daily tasks. This is one of the critical factors required by the clinical environment, which leads to an increase in the acceptance rate.

The current study has several implications for different aspects of digital transformation in healthcare, particularly the adoption of CDSS in Saudi Arabia. First, our results show that usability positively influences the intention to use CDSS. Therefore, developers and policymakers need to use user feedback in the design usability features of CDSS. Second, the results indicate that the system’s availability significantly affects user acceptance of CDSS. Hence, IT infrastructure must ensure a high level of system availability. Third, the results show that physicians should be allowed to override the CDSS recommendations and provide supporting evidence. Therefore, CDSS designers must consider providing the override option. Fourth, the results show that training sessions have a positive influence on the intention to use CDSS. Therefore, it is essential to consider the suitable training needed before implementing CDSS. Finally, the results show that technical support provided by Information Systems is a critical factor for CDSS acceptance.

This research has some limitations. First, the study used a cross-sectional survey questionnaire to collect the data sample, making it challenging to investigate causal relationships [31] among the research model constructs. Future research might use a longitudinal survey questionnaire to collect the data over an extended period to obtain more reliable interpretations.

Second, our study only used the quantitative method. Mixing quantitative and qualitative methods might be an excellent future research direction to validate the results of this study.

Third, the subjects of this study are from five hospitals, but not all hospitals are equally represented in the sample. This might lead to some bias in the data set. Although the five hospitals are under one administration and share the same policies, regulations, standards, salary scales, benefits, facilities, and working hours, future research studies should consider the uniform representation of all five hospitals in the data set to avoid any chance for bias.

## 7. Conclusions

This study found predictive factors influencing healthcare practitioners’ intention to use BESTCare 2.0 to provide healthcare services and assist them in decision making based on the developed model. As expected, the study found that the ten used variables are critical and predictive factors in CDSS acceptance. The results confirmed that the variables played an essential role in the outcomes of CDSS acceptance. The results from hypotheses testing show that the system’s usability and availability, medical history accessibility, task impact, and task technology fit positively correlate with user intention to use.

This study provides prudence pertaining to healthcare facilities and their higher management to adopt CDSS. The study proved the positive impact of the implementation of CDSS on healthcare quality and saving financial expenses. Another vital contribution to the literature and Saudi healthcare facilities is the development of a new acceptance model with specific factors and variables. Such a model will facilitate the measurement of users’ acceptance and behavior toward medical systems, which will shorten the required time to choose a new medical system for any organization and provide the factors of success and lessons learned. In fact, the analysis of acceptance capability is a very important step toward the successful adoption and implementation of such CDSS [87].

## Figures and Tables

**Table 1:** Descriptive statistics.

Measure	Item	Percent
Gender	Male	85%
	Female	15%
Age	30 years or less	25%
	Between 31 and 40	38.3%
	Between 41 and 50	30%
	More than 50 years	6.7%
Job title	Consultant	18.3%
	Resident	11.7%
	Clinical pharmacist	20%
	Pharmacist	50%
Hospital	Riyadh	56.7%
	Jeddah	1.7%
	Ahsa	31.7%
	Dammam	3.2%
	Madinah	6.7%

**Table 2:** Reliability results.

Construct	No of Items	Cronbach’s Alpha	Composite Reliability
System’s usability	2	0.78	0.87
System’s availability	2	0.85	0.88
Medical history	3	0.86	0.95
Training sessions	2	0.90	0.92
User support	3	0.79	0.93
Override justification	2	0.60	0.73
Task impact	2	0.84	0.92
Task-Technology Fit	3	0.95	0.98
Intention to use	3	0.92	0.94
Net Benefit	3	0.93	0.97

**Table 3:** Convergent validity.

Variable Constructs	Composite Reliability	AVE
System’s usability	0.87	0.65
System’s availability	0.88	0.76
Medical history	0.95	0.66
Training sessions	0.92	0.82
User support	0.93	0.56
Override justification	0.73	0.50
Task impact	0.92	0.73
Task-Technology Fit	0.98	0.87
Intention to use	0.94	0.74
Net benefit	0.97	0.84

**Table 4:** Discriminant validity.

	TT	IU	SU	NB	MH	TS	US	OJ	TI	SA
**TT**	**0.931**									
**IU**	0.650	**0.860**								
**SU**	0.790	0.669	**0.806**							
**NB**	0.682	0.767	0.806	**0.916**						
**MH**	0.744	0.753	0.720	0.777	**0.812**					
**TS**	0.652	0.364	0.671	0.476	0.652	**0.906**				
**US**	0.655	0.449	0.518	0.533	0.500	0.716	**0.748**			
**OJ**	0.454	0.217	0.452	0.302	0.204	0.315	0.228	**0.707**		
**TI**	0.850	0.630	0.651	0.772	0.723	0.616	0.530	0.341	**0.854**	
**SA**	0.720	0.601	0.800	0.676	0.740	0.657	0.435	0.313	0.727	**0.872**

**TT**: Task-Technology fit. **IU**: Intention to Use. **SU**: Systems Usability. **NB**: Net Benefit. **MH**: Medical History. **TS**: Training Sessions. **US**: User Support. **OJ**: Override Justification. **TI**: Task Impact. **SA**: System Availability.

**Table 5:** Factor loadings.

	SA	SU	MH	TS	US	OJ	TI	TT	IU	NB	*p* Value
SA1	0.788										<0.001
SA2	0.947										<0.001
SU1		0.784									<0.001
SU2		0.821									<0.001
MH1			0.882								<0.001
MH2			0.808								<0.001
MH3			0.734								<0.001
TS1				0.870							<0.001
TS2				0.942							<0.001
US1					0.858						<0.001
US2					0.795						<0.001
US3					0.544						<0.001
OJ1						0.750					<0.001
OJ2						0.596					<0.001
TI1							0.895				<0.001
TI2							0.809				<0.001
TT1								0.937			<0.001
TT2								0.915			<0.001
TT3								0.941			<0.001
IU1									0.911		<0.001
IU2									0.904		<0.001
IU3									0.867		<0.001
NB1										0.883	<0.001
NB2										0.904	<0.001
NB3										0.958	<0.001

**TT:** Task-Technology fit. **IU:** Intention to Use. **SU:** Systems Usability. **NB:** Net Benefit. **MH:** Medical History. **TS:** Training Sessions. **US:** User Support. **OJ:** Override Justification. **TI:** Task Impact. **SA:** System Availability.

**Table 6:** Hypothesis conclusion.

Hypothesis	Finding	Conclusion
**H1.** System’s usability has a positive influence on intention to use.	Yes: (β = 0.35, *p* < 0.001)	Supported
**H2.** System’s availability has a positive influence on intention to use.	Yes: (β = 0.34, *p* < 0.001)	Supported
**H3.** Medical history accessibility has a positive influence on intention to use.	Yes: (β = 0.45, *p* < 0.001)	Supported
**H4.** Task impact has a positive influence on intention to use.	Yes: (β = 0.32, *p* < 0.001)	Supported
**H5.** Task-Technology Fit has a positive influence on intention to use.	Yes: (β = 0.39, *p* < 0.001)	Supported
**H6.** Override justification has a positive influence on intention to use.	Yes: (β = 0.13, *p* < 0.001)	Supported
**H7.** Training sessions has a positive influence on intention to use.	Yes: (β = 0.16, *p* < 0.001)	Supported
**H8.** User support has a positive influence on intention to use.	Yes: (β = 0.23, *p* < 0.001)	Supported
**H9.** Intention to use has a positive influence on Net benefit.	Yes: (β = 0.57, *p* < 0.001)	Supported

**Table 7:** Total of alerts by type.

Alert	Total	Percentage
Class/Group	1005	2.17%
Contraindicated	2622	5.67%
Cross-Reaction	2145	4.64%
Drug-Drug Severity Major	18,447	39.92%
Drug-Drug Severity Moderate	9	0.02%
Ingredient	72	0.16%
Not recommended	1	0.001%
Professional Intervention Required	3210	6.95%
Professional Review Suggested	13	0.03%
Single Dose Maximum	18,688	40.44%
Grand Total	46,212	100%

**Table 8:** Alerts and override by visit type.

	Total			Percentage		
Physician’s Override?	ER	Inpatient	Outpatient	ER	Inpatient	Outpatient
No	6234	17,575	10,350	78.14%	74.18%	71.17%
Yes	1744	6116	4193	21.86%	25.82%	28.83%

## Data Availability

Not applicable.

## Appendix A

Constructs and items
System’s usability
SU1. This system is easily used [52].
SU2. This system has a quick response [52].
System’s availability
SA1. This system is always available for business [54].
SA2. This system launches and runs right away [54].
Medical history accessibility
MH1. Information provided in the system is up-to-date [55].
MH2. Information provided in patient’s profile is easy to understand [55].
MH3. The system provides all patient relevant information necessary to fulfill my needs [55].
Training sessions
TS1. I consider the training used for this system is adequate [52].
TS2. I completely accept the training approach of this system [52].
User support
US1. The provider (ISID) is very sophisticated with this system [52].
US2. The provider (ISID) of this system is able to rapidly solve the operating problems [52].
US3. Generally, the provider (ISID) of this system treats its customers with courtesy [52].
Override justification
OJ1. If I override a drug alert, it is because the risk of the drug (or drug combination) is acceptable after considering the therapeutic benefit [60].
OJ2. If I override a drug alert, it is because this drug alert is not clinically important for the given patient [60].
Task impact
TI1. The system helps me to meet patient’s needs [56].
TI2. The system allows me to accomplish more work than would otherwise be possible [56].
Task-Technology Fit
TT1. In my opinion, the system’s functions are suitable for helping me complete my task [57].
TT2. In my opinion, the system’s functions are enough to help me complete my task [57].
TT3. In my opinion, the system’s functions are fit for the requirements of my work or coursework [57].
Intention to use
IU1. I am willing to use the system as an aid to help with my decisions about which medication to prescribe [62].
IU2. I am willing to let the system assist me in deciding which medication to prescribe [62].
IU3. I am willing to use the system as a tool that suggests to me a number of medications from which I can choose [62].
Net benefit
NB1. The system has changed my job significantly [63].
NB2. The system has reduced the time it takes to support decision making [63].
